# Advances in the Structure, Function, and Regulatory Mechanism of Plant Plasma Membrane Intrinsic Proteins

**DOI:** 10.3390/genes16010010

**Published:** 2024-12-25

**Authors:** Xueting Li, Yirong Guo, Qiuping Ling, Zhejun Guo, Yawen Lei, Xiaomin Feng, Jiayun Wu, Nannan Zhang

**Affiliations:** 1 Guangdong Sugarcane Genetic Improvement Engineering Center, Institute of Nanfan & Seed Industry, Guangdong Academy of Sciences, Guangzhou 510316, China; lxt19910226@163.com (X.L.); jiayunng@163.com (J.W.); 2 Zhanjiang Research Center, Institute of Nanfan & Seed Industry, Guangdong Academy of Sciences, Zhanjiang 524300, China; 3 National Engineering Research Center for Sugarcane, Fujian Agriculture and Forestry University, Fuzhou 350002, China; 4 Rice Research Institute, Guangdong Academy of Agricultural Sciences, Guangzhou 510640, China

**Keywords:** plasma membrane intrinsic proteins, structure and function, molecular mechanism, regulatory mechanism

## Abstract

Plasma membrane intrinsic proteins (PIPs), as members of the aquaporin (AQPs) family, can transport not only water but also urea, CO_2_, H_2_O_2_, metal ions, and trace elements. They are crucial for maintaining water balance, substance transport, and responding to various stresses. This article delves into the structure, function, response mechanism, molecular mechanism, and regulatory mechanism of PIPs as a result of biological and abiotic stresses. It also summarizes current research trends surrounding PIPs and highlights potential research directions for further exploration. The aim is to assist researchers in related fields in gaining a more comprehensive understanding and precise insight into the advancements in PIP research.

## 1. Introduction

Plant plasma membrane intrinsic proteins (PIPs) constitute a vital protein family located on the cytoplasmic membrane of plants. These proteins possess a highly specific channel structure for the transport of water molecules, enabling the efficient transmembrane movement of water and playing a crucial role in regulating water absorption, transport, and maintaining the osmotic pressure balance within plant cells [1,2]. Plant cells can rapidly absorb and transport water through the water channel formed by PIP proteins [2]. They are essential for plants’ responses to varying water conditions, such as drought and salt stress; for instance, under drought conditions, in order to reduce water loss, plants will change the water permeability of cells by regulating the expression of PIPs [3]. In saline environments, they help plant cells balance the osmotic pressure inside and outside the cells [4]. Moreover, PIPs play an important regulatory role in the whole process of plant growth and development. In the stage of seed germination, PIP proteins are involved in regulating the water absorption of seed cells and providing the necessary water conditions for seed germination. In the vegetative growth stage of plants, the expression level of PIPs is closely related to cell elongation and division [5]. The expression of PIPs is regulated by many factors. At the transcriptional level, some transcription factors in plants can bind to the promoter region of PIPs and regulate its transcriptional activity. At the post-translational level, the activity and stability of PIP proteins are also regulated by a variety of modifications. For example, phosphorylation modification can change the conformation and function of PIP proteins and affect their ability to transport water and other substances [6]. Thus, an in-depth study of the structure and function of aquaporins is of great significance for understanding the water relationships and stress resistance mechanisms in plants. Additionally, it holds promise for enhancing crop water use efficiency and stress resilience in agricultural production, providing a crucial theoretical foundation and potential genetic resources for the cultivation of superior crop varieties that can better adapt to diverse environmental conditions.

## 2. Structure and Classification of PIPs

### 2.1. Origin, Classification, and Structure of AQPs

Aquaporins (AQPs), a widely distributed membrane channel protein in organisms, transport water as well as other molecules across various cell membranes. They were first discovered by Agre, who isolated a 28 kD molecule with hydrophilic amino acids from human red blood cell membranes, named CHIP28 (later renamed AQP1), and subsequently confirmed its function in water transport across membranes, thus beginning the human understanding of AQPs [7]. The discovery of AQP1 sparked a wave of isolating and identifying AQPs. Within a few years, many homologous genes of AQPs had been isolated in plants, animals, bacteria, and yeasts. AQPs can be found in plastids, vacuoles, the endoplasmic reticulum, and the plasma membrane [3], and based on their amino acid sequence homology as well as their subcellular localization, four major subfamilies can be identified: small basic intrinsic proteins (SIPs), Nodulin 26-like intrinsic proteins (NIPs), tonoplast intrinsic proteins (TIPs), and plasma membrane intrinsic proteins (PIPs) [8]. Subsequently, proteins homologous to bacterial glycerol channel proteins, including X-intrinsic proteins (XIPs), hybrid intrinsic proteins (HIPs), and Glp F-like intrinsic proteins (GIPs), were also identified [9].

**Figure 1:**
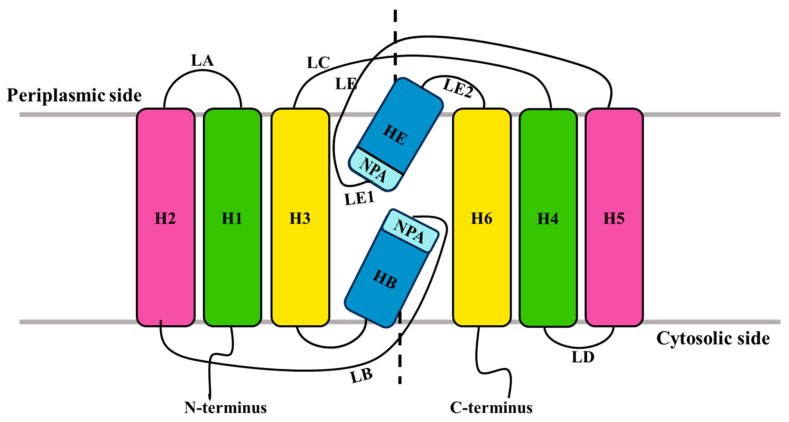
Membrane topology diagram of AQPs. This image is from Luang (2017) Structural Basis of the Permeation Function of Plant Aquaporins, and is used in its original form [13].

The basic structure of aquaporin membrane channel proteins is a single polypeptide chain consisting of two homologous repeats, each containing a highly conserved Asn-Pro-Ala (NPA) amino acid [10]. The topology indicates that each monomer consists of two short α-helices (HB and HE) and six tilted (crossing angles between 25°and 40°) transmembrane α-helices (H1-H3 and H4-H6), with the amino acid chain forming five loop structures on either side of the membrane (A, B, C, D, E). Topological studies reveal that loops B and D, as well as the carboxylic (COOH–, abbreviated as C-) and amino (NH_2_-, abbreviated as N-) termini, are located intracellularly, while loops A, C, and E are located extracellularly. The two repeats exhibit significant amino acid sequence similarity and are arranged in a 180° center-symmetric manner on the membrane. Loops B and E are hydrophobic and contain the conserved Asn-Pro-Ala (NPA) repeat motif, which is a highly conserved characteristic sequence shared by members of this protein family. Nearly all the sequenced MIP genes possess this domain motif [11] (as shown in Figure 1). In contrast, the N-terminus and C-terminus show greater variability. The deletion of eight amino acids in the C-terminus of the spinach aquaporin intrinsic protein SoPIP2;1 does not affect its activity, indicating that the C-terminus is not directly involved in water transport [12].

### 2.2. Conserved Regions and Classification of PIPs

(a) The Conserved Regions of PIPs: In higher plants, two highly conserved regions exist in PIPs: GGGANXXXXGY and TGINPARSLGAA, located in the C and E loops, respectively [13], which may be related to the specificity of the PIPs’ function.

(b) Classification and differences in PIP subfamilies: PIPs are divided into two subclasses, PIP1 and PIP2 [14], with significant differences in water transport activity between the two subgroups. The PIP2 protein has been described as a good water channel protein in the African clawed frog expression system, while PIP1 members showed lower water channel activity in this expression system [15], which is consistent with experimental results in rice [16]. Variations in the specific amino acid sequences of the NPA motifs and the six transmembrane alpha helices of PIP1 and PIP2 proteins contribute to differences in water permeability [14]. Furthermore, the PIP2 protein is characterized by a longer C-terminal extension and a shorter N-terminal extension compared to the PIP1 protein, which includes putative phosphorylation sites [17]. Studies have shown that the phosphorylation of serine residues regulates the water transport activity of the spinach PIP2 member PM28A [18]. According to Johanson’s nomenclature, the PIP1 subgroup comprises five water channel protein members named PIP1;1 to PIP1;5, while the PIP2 subgroup consists of eight plasma membrane water channel protein members named PIP2;1 to PIP2;10 [5].

(c) PIP expression across species: Currently, PIP gene families have been identified in several plants, including *Arabidopsis thaliana* (13 members) [19], *Oryza sativa* (11 members) [20], *Cicer arietinum* (9 members) [21], *Citrus sinensis* (11 members) [22], and others as shown in Table 1 [23,24,25,26,27].

## 3. The Function of PIPs

### 3.1. Physiological Function

The unique structure of PIPs is crucial for the different physiological activities of plants, including mediating the rapid transmembrane transport of water [6,28,29,30], participating in stomatal movements [31]; involvement in the transport of CO_2_ within leaves [32]; regulating the absorption of neutral molecules (glycerol, NH_3_, urea) and nutrients (B, Si) [33,34,35,36]; and the transport and signaling of H_2_O_2_ [37]. Meanwhile, PIPs play physiological roles under the influence of various factors such as pH, Ca^2+^, heavy metal ions (Hg^2+^, Ag^+^), phosphorylation [13,38,39,40,41], as well as plant hormones (abscisic acid, gibberellins, and ethylene), salt stress, drought, and infection by pathogenic microorganisms, which can induce the expression of PIP proteins [27,42,43]. Most plant organs, such as seeds, fruits, flowers, stems, leaves, roots [44,45,46], pollen, anthers [47,48,49,50], and their specialized cells such as guard cells [51,52], contain PIPs.

### 3.2. Function Under Abiotic Stress

Under field conditions, crops face a wide range of biotic and abiotic challenges. Environmental factors such as high salinity, unfavorable hot or cold temperatures [53,54,55,56], and biological stresses such as diseases, are the main reasons for global crop yield losses [57]. The responses of plants to various stressors are quite complex, with multiple mechanisms being simultaneously activated to restore cellular homeostasis and enhance survival [58]. When facing biotic and abiotic stresses, the water balance of cells, tissues, and the entire plant body is disrupted. Therefore, under stress conditions, the regulation of AQPs becomes crucial for cells to maintain homeostasis [59]. Previous studies have shown that *PIP* genes can regulate gene expression levels under various stress conditions, thereby modulating substance accumulation [60,61]. They can also interact with various stress-related proteins to collectively regulate plant water and osmotic balance, enhancing plant stress resistance and adaptability [61]. Analyzing and summarizing the responses and expressions of plasma membrane aquaporin genes under different environmental conditions is of great significance to better understanding their roles in plants under various physiological conditions and for guiding future research.

#### 3.2.1. Response to Drought

Water is essential for plant survival. When the water content in the soil and air decreases, this affects the photosynthesis of plants and thus normal plant growth [62]. AQPs are important channels that control the movement of water. Therefore, studying how AQPs respond to drought conditions is of great significance. Jang et al. reported the expression patterns of *AtPIPs* under abiotic stress, showing that, in response to drought stress, most *AtPIPs* were significantly downregulated in the roots [63]. The expression of *MaPIP2-3* and *MaPIP2-7* was significantly induced after osmotic treatment in BaXi Jiao, while the expression of *MaPIP2-6* decreased. However, the expression of *MaPIP2-6* significantly increased in Fen jiao, indicating that, in different plants, the expression of *PIP* family members varies in response to drought conditions [64]. The movement of water mediated by PIPs, both intercellular and intracellular, is crucial for enhancing bananas’ resistance to osmotic stress. Overexpression of *PIP* family genes positively regulates plant drought tolerance by increasing the content of osmotic regulatory substances and antioxidant enzyme activity, reducing ion leakage and lipid peroxidation to enhance plant drought tolerance [65,66]. For example, the transgenic banana plants Dhn-*MusaPIP1;2* and Ubi-*MusaPIP1;2* exhibited stronger drought resistance compared to the control, along with positive biochemical indicators and quicker recovery from stress damage, possibly due to an increase in the number of water channel proteins and higher concentrations of compatible solutes (free proline) to improve cell water levels, thereby reducing damage to cell membranes (e.g., chloroplast membranes) as a result of abiotic stress [67]. In Arabidopsis, the heterologous expression of *MaPIP1;1* could enhance drought tolerance by maintaining osmotic balance, improving ion distribution, and reducing membrane damage [68]. Additionally, under drought conditions, the overexpression of *PvPIP29* in transgenic rice presented more significant changes in expression than most other PIP2 members, indicating the coordinated transcriptional regulation of *PIP2* subfamily genes [65]. According to Hu et al., overexpressing *TaAQP8*, a gene of the PIP1 subfamily, in transgenic tobacco enhanced the plants’ resistance to drought [69]. In rice protoplasts, *OsPIP2;2* significantly enhanced H_2_O transport and drought responses, inducing an increase in proline and polyamine concentrations (both physiological indicators of drought tolerance). The expression of drought pathway marker genes significantly increased, while the expression of related genes was strongly inhibited when *OsPIP2;2* was silenced. Moreover, *OsPIP2;2* was involved in maintaining the integrity of cell membranes, thereby protecting rice cells from the leakage of electrolytes as a result of drought conditions. Altogether, the findings indicate that *OsPIP2;2* acts a major facilitator of water transport associated with plant drought resistance [70]. In tomato plants, plasma membrane aquaporins (*SlPIP2;1, SlPIP2;7*, and *SlPIP2;5*) play a crucial role in water uptake by maintaining osmotic balance and improving water content [71]. Overall, members of the *PIP* gene family actively respond to drought stress, with the members of the *PIP2* subfamily playing a more positive role in plant drought resistance than the *PIP1* subfamily members, possibly due to the better water channel activity exhibited by *PIP2*.

#### 3.2.2. Response to Low Temperature

Low temperatures can inhibit the physiological and biochemical activities of plants, such as reducing transpiration, lowering respiratory function, and disrupting physiological balance, leading to the cessation of plant life activities or death. Therefore, studying the response mechanism of the *PIP* gene family to low-temperature stress and identifying potentially cold-resistant *PIP* genes is of great significance for plant production.

Low-temperature treatment was shown to downregulate the expression of most *AtPIPs* in the roots, while salt treatment led to their upregulation [63]; Arabidopsis that had been subjected to cold stress conditions was studied using real-time quantitative reverse transcription PCR technology to determine the expression levels of a gene family encoding 13 *PIPs*. In this case, the cold treatment downregulated most *PIP* genes, with only the *AtPIP2;5* gene being upregulated [63]. In a different study, various AQP subtypes of cold-tolerant and cold-sensitive rice varieties were compared to comprehensively analyze differences in their expression and regulation, with the results revealing that *OsPIP1;1* and *OsPIP1;2* were closely related to cold tolerance [72]. The overexpression of *MdPIP2;5a* and *MdPIP2;5b* genes in Arabidopsis increased the tolerance of transgenic Arabidopsis to cold stress [73]. Li et al. found that the overexpression of almond *PaPIP1-2* in yeast strains resulted in a higher protein content and better cold resistance. Similarly, when overexpressed in Arabidopsis, *PaPIP1-2* enhanced the growth of transgenic plants under cold stress by lowering the level of malondialdehyde (MDA), increasing proline (Pro) accumulation, and increasing superoxide dismutase (SOD) activity [74]. In a study on the seasonal cold adaptation (CA) of rhododendron, Peng et al. found that the expression of *RcPIP2;1* and *RcPIP2;2* decreased as leaf freezing tolerance (FT) increased from −7 °C to approximately −50 °C. The overexpression of *RcPIP2s* in Arabidopsis and comparison with wild-type (WT) plants showed that the constitutive FT and CA abilities of *RcPIP2* plants were significantly reduced, possibly due to their lower ability to resist freeze drying [75]. Xu et al. subjected seedlings of two rice varieties adapted to different temperatures to cold storage at 7 °C, followed by recovery at 28 °C, and identified the mRNA expression profiles of all 11 *PIPs* using RT-qPCR. They found that most *PIP* genes were downregulated under low temperature and recovered at high temperature [76]. The banana *MaPIP1;2* gene was also shown to positively influence tolerance to cold stress, and its overexpression increased the tolerance of transgenic material [67]. Similarly, the wheat aquaporin protein *TaAQP7* conferred stronger cold tolerance to transgenic tobacco compared to non-transgenic tobacco [77,78,79,80].

#### 3.2.3. Response to Salt Ion Stress

Soil salinity, which influences around 20% of the world’s arable land, represents a major challenge for agricultural planners and scientists. Indeed, salt stress negatively affects the physiological processes governing plant growth, thereby decreasing plant productivity [8]. Water availability is directly impacted by salt stress, making the study of AQPs crucial in the context of salt stress response. The MzPIP2;1 protein plays a crucial role in the radial movement of water, while controlling water absorption and utilization to maintain water transport in transgenic Arabidopsis under salt stress conditions, thereby improving survival rates [80]. In transgenic rice, enhanced salt tolerance was observed following moderate overexpression of *OsPIP1;1* [81]. Similarly, in bananas, the constitutive and stress-induced overexpression of *MusaPIP2;6* could increase their resistance to salt stress [82]. Overexpressing *MdPIP2;5a* and *MdPIP2;5b* genes in Arabidopsis enhanced the transgenic plants’ sensitivity to salt stress [73]. While studying miRNAs and their target genes that were differentially expressed under salt stress, Roohollah and colleagues identified significant differences in the expression profile of *miR1118* and its predicted target gene *TaPIP1;5* among wheat genotypes. *MiR1118* primarily regulates membrane damage, ion homeostasis, and wheat water status through *TaPIP1;5* [83]. Hamza and colleagues functionally validated the salt stress response of *CmoPIP1-4* knockout mutants in pumpkins, showing that the expression of *CmoPIP1* was responsive to salt stress in leaves and roots, mainly manifested in phenotypic (weakened plant health state), physiological (decreased enzyme activity), and molecular (reduced expression of immune-related genes) aspects; the overexpression of *CmoPIP1-1-4* conferred salt tolerance to yeast [84]. Zhou et al. obtained and identified the function of the *ZmPIP1;1* in drought and salt tolerance in *A*. *thaliana*: NaCl treatment induced *ZmPIP1;1* expression in roots and leaves, and compared to the wild type, transgenic Arabidopsis plants also exhibited enhanced tolerance to salt stress. Indeed, plants overexpressing ZmPIP1;1 exhibited higher levels of proline, lower levels of reactive oxygen species (ROS)-scavenging enzyme activity (e.g., malondialdehyde, hydrogen peroxide, and superoxide), and higher activities of antioxidant enzymes (e.g., superoxide dismutase and catalase) than the wild type in response to salt and drought stress [85]. Similarly, under salt stress conditions, mRNA expression analysis in citrus under abiotic stress showed a significant upregulation of *CsPIP1;1* in roots [86]; in grapes, salt stress increased the transcription of *PIP2;1* [87]. Most *StPIPs* in potatoes (*Solanum tuberosum*) showed differential expression under salt stress [88]. The overexpression of *EsPIP2;1* in salt cress (*Eutrema salsugineum*) enhanced the salt stress tolerance of *Arabidopsis* [89]. In addition, the overexpression of buffalo gourd (*Cucurbita ficifolia*) *CfPIP2;1* and cucumber *CsPIP1;1* in *Arabidopsis* increased the germination rate of seeds subjected to high salt stress [90], thus indicating that *AQPs* can enhance the salt tolerance of transgenic plants. However, some studies have shown that the overexpression of *AQP* genes can reduce salt sensitivity in plants; for instance, overexpressing *HvPIP2;1* in rice can decrease salt tolerance [91].

#### 3.2.4. Response to Pseudo Metal Ions

In biological systems, the roles of metalloids vary from essential B to beneficial Si, and to toxic As and Sb. Significant research has recently been focused on identifying and assessing the expression of metalloid transporters that assist in the uptake of metalloids from the soil into plant roots and their translocation to shoots and seeds within plant tissues. Water channel proteins have gained more attention as key players in metalloid transport in plants over the past decade. Therefore, investigating the distribution and regulatory mechanisms of PIP genes in plants with respect to metalloid elements contributes to enriching our understanding of the function of plant plasma membrane water channel proteins.

In yeast transport experiments, the overexpression of barley *HvPIP1;3* and *HvPIP1;4* in yeast cells confers a boron transport function [92]; after high boron treatment, the increased transcription of *OsPIP2;6* and *OsPIP1;3* was noted in rice roots, participating in boron influx and efflux, indicating that *OsPIP1;3* has a bidirectional boron transport function. Under boron stress, excess boron flows quickly from the roots to the external culture medium, while the overexpression of *OsPIP2*;6 and *OsPIP1;3* yielded high boron tolerance in transgenic Arabidopsis. When two water channel protein inhibitors NaN_3_ and AgNO_3_ are added separately, the tolerance phenotype of transgenic Arabidopsis overexpressing *OsPIP1;3* and *OsPIP2;6* under high boron conditions is significantly suppressed. In addition, under high boron treatment, Arabidopsis downregulates the expression of water channel proteins such as *AtPIP1;2*, *AtPIP2;1*, and *AtPIP2;2* in roots and leaves, reducing water transport in tissues, which also helps reduce water flow to the shoots, preventing excess boron accumulation in plant tissues [93]. Under high boron conditions, NIPs, TIPs, and PIPs are involved in reducing boron accumulation in plant tissues, so these water channel proteins play important roles in enhancing plant tolerance to boron deficiency or excess. This may reduce the intercellular movement of water in plant tissues, thereby reducing water flux to the stems. All these changes in plant water balance under B toxicity may be mechanisms to prevent excess boron accumulation in plant tissues [94].

Ahmed et al. found a significant reduction in arsenic accumulation in rice stems by inhibiting the expression of *OsPIP2;6* through an RNAi-mediated approach. Conversely, the overexpression of *OsPIP2;6* led to an increase in arsenic accumulation in the aboveground parts of rice, indicating the crucial role of *OsPIP2;6* in the transport of arsenic from roots to stems, reducing arsenic accumulation in rice [95]. In addition, it has been shown that three PIPs in rice, namely *OsPIP1;3*, *OsPIP2;4*, and *OsPIP2;7*, were involved in the transport of boron arsenate (AsIII) and provided tolerance to AsIII and boron toxicity [96,97]. Mahsa et al. observed that *Arabidopsis AtPIP2;2* encodes an intrinsic plasma membrane protein and shows better arsenite (As(III)) tolerance when highly expressed in Arabidopsis expressing NtCyc07. The overexpression of *AtPIP2;2* increased the tolerance of yeast, and Arabidopsis overexpressing As(III) reduced As(II) levels in yeast and yielded the opposite results in knockout experiments [98].

The *PIP* gene directly or indirectly participates in the regulation of various metal elements in cells, playing a key role in plant detoxification and stress resistance.

### 3.3. Function Under Plant Diseases

**Figure 2:**
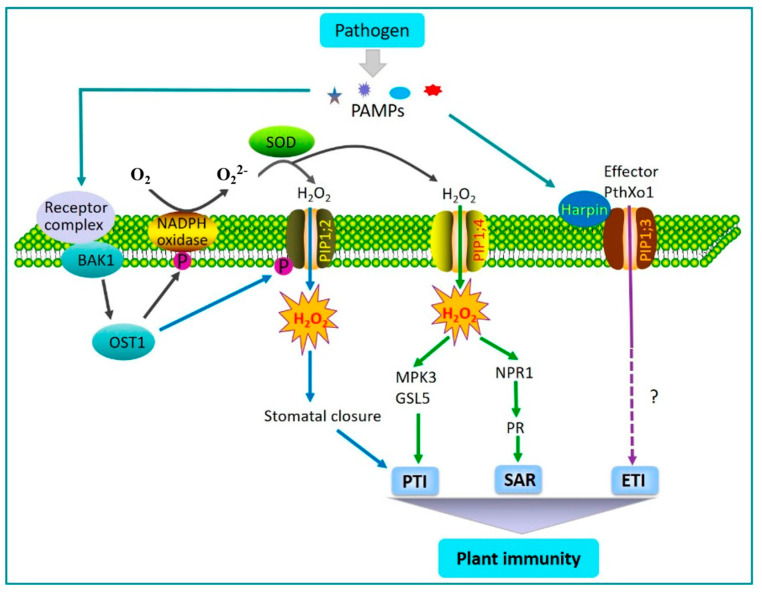
A working model for the involvement of aquaporins in mediating plant immunity. The image is from Li (2020), Roles of aquaporins in plant-pathogen interaction. and is used in its original form [101].

Plants in nature are inevitably attacked by pathogens such as viruses, bacteria, fungi, etc., leading to severe impacts on economic crops and causing economic losses [99,100]. However, plants also have their own immune system to defend against pathogen invasion, with the first line of defense mainly relying on the function of biomolecules such as membrane proteins [101] (Figure 2). In addition, plants themselves undergo various changes, including the production of reactive oxygen species, ion flux, changes in gene expression levels, etc. More and more evidence indicates that PIPs, as channel proteins in plants, are crucial for interactions between plants and pathogens, participating in plant immunity or pathogen pathogenicity [99].

The outbreak of ROS is a marker for plants to successfully recognize pathogens or pathogen-associated molecular patterns (PAMPs) [102]. As a significant signaling molecule in living organisms, H_2_O_2_ serves as a primary component of ROS and participates in various physiological and pathological processes [103]. PAMP-induced H_2_O_2_ usually accumulates in the extracellular space of plants. PIPs mediate the transport of extracellular H_2_O_2_ into cells, interacting with immune pathways such as pattern-triggered immunity (PTI) and systemic acquired resistance (SAR), regulating plant disease resistance [104]. Studies have shown that both the individual and co-expression of *AtPIP1;4* and *AtPIP2;4* can promote the transport of extracellular H_2_O_2_ into cells and play an essential role in signaling pathways such as PTI and SAR, enhancing resistance to Pseudomonas syringae. The co-expression of *AtPIP1;4* and *AtPIP2;4* can positively influence the plant’s immune response through the PTI pathway [105,106]. *AtPIP2;1* mediates the transport of H_2_O and H_2_O_2_ across the plasma membrane of guard cells, triggering the stomatal closure induced by abscisic acid and pathogens, to defend against pathogen invasion [52]. Plasma membrane intrinsic protein *AtPIP1;4* interacts with harpin protein Hpa1 produced by *Xanthomonas oryzae* pv. *oryzae*, thereby regulating Hpa1-induced plant growth promotion [107]. By regulating the transmembrane transport of H_2_O_2_ and activating MAPK cascade pathways, promoting callus deposition, and inducing PR gene expression, the *OsPIP2;2* and *TaPIP2; 10* genes play an important role in responding to the PTI pathway to resist pathogen invasion [108,109]. The *OsPIP2;2* gene enhances rice’s basal immune response by regulating *OsmaMYB* nuclear translocation. This study found that bacterial blight or sheath blight infection in rice causes the upregulation of *OsPIP2;2* expression, and *OsPIP2;2* positively regulates rice’s resistance to bacterial blight, sheath blight, and blast disease. In rice, *OsPIP1;3* is involved in plant immunity by mediating the transport of effector PthXo1. The infection of rice by bacterial blight or sheath blight leads to the formation of an Hpa1-*OsPIP1;3* complex, where the bacterial harpin protein Hpa1 hijacks *OsPIP1;3* to transfer the PthXo1 effector from bacterial cells to rice cells, promoting pathogen infection in rice and affecting rice’s disease resistance [110]. Harpin protein Hpa1 from *Xanthomonas oryzae* pv. *oryzae* interacts with the *OsPIP1;2* protein, suggesting its involvement in rice’s immune response to pathogenic bacteria [111]. We listed PIP genes involved in biotic and abiotic stresses in Table 2.

## 4. The Expression and Regulatory Mechanism of PIP

### 4.1. The Specificity of PIP Expression Mechanisms in Different Organizations

**Figure 3:**
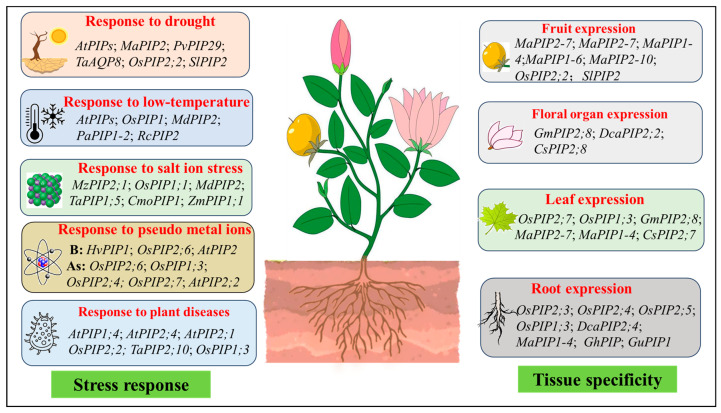
Biological function and tissue specificity of PIPs. *AtPIPs* [63], *MaPIP2* [64], *PvPIP29* [65], *TaAQP8* [69], *OsPIP2;2* [70], and *SlPIP2* [71] can respond to low temperatures. *AtPIPs* [63], *OsPIP1* [72], *MdPIP2* [73], *PaPIP1-2* [74], and *RcPIP2* [75] can respond to salt ion stress*. MzPIP2;1* [80], *OsPIP1;1* [81], *MdPIP2* [82], *TaPIP1;5* [83], *CmoPIP1* [84], *ZmPIP1;1* [85], and *CsPIP1;1* [86] can respond to salt ion stress. *HvPIP1* [92], *OsPIP2;6*, *OsPIP1;3*, and *AtPIP2* [94] can respond to pseudo metal ions (B). *OsPIP2;6* [95], *OsPIP1;3*, *OsPIP2;4*, *OsPIP2;7* [96,97,98], and *AtPIP2;2* [99] can respond to pseudo metal ions (As)*. AtPIP1;4*, *AtPIP2;4* [52,106,108], *AtPIP2;1* [107], *OsPIP2;2* [109], *TaPIP2;10* [110], and *OsPIP1;3* [111] can respond to plant diseases. *MaPIP2-7*, *MaPIP2-10*, *MaPIP1-4*, and *MaPIP1-6* [68] can respond to fruit expression. *GmPIP2;8* [115], *DcaPI3*,114], *GmPIP2;8* [115], *MaPIP2-7, MaPIP1-4* [68], and *CsPIP2;7* [117] can respond to leaf expression. *OsPIP2;3*, *OsPIP2;4*, *OsPIP2;5*, *OsPIP1;3* [113,114], *DcaPIP2;4* [116], *MaPIP1-4* [68], *GhPIP* [60], and *GuPIP* [118] can respond to root expression.

The expression levels and patterns of various PIPs in different plant organs vary, showing constitutive expression at high/low levels or expression only in certain specific tissues (Figure 3). It was found that most rice tissues contained *OsPIP1;1*, *OsPIP1;2*, *OsPIP2;1*, *OsPIP2;2*, and *OsPIP2;6*. *OsPIP2;3*, *OsPIP2;4*, and *OsPIP2;5* were mainly expressed in roots, *OsPIP2;7* was mainly expressed in leaves, and *OsPIP1;3* was expressed in both roots and leaves [112,113]. In soybean, *GmPIP2;8* was mainly expressed in leaves and floral organs [114]. *DcaPIP2;2* was only expressed in floral organs, and *DcaPIP2;4* was only expressed in roots [115]; *MaPIP1-4* and *MaPIP2-7* were highly expressed in the roots, leaves, and fruits of Baxijiao (BXJ) and Fenjiao (FJ), while a high abundance of *MaPIP1-6* and *MaPIP2-10* transcripts were noted at all stages of BXJ and FJ fruit development and maturation, promoting fruit development [67]. Reuscher et al. conducted whole-genome identification of the AQPs gene family, performed tissue-specific expression analysis on the identified PIP gene, and found that *SlPIP1;1* had a strong expression signal in the root tissue, but was not expressed in the stem and leaf tissues [116]. Different genotypes of Musa acuminatum grown under water stress also exhibit differential expression patterns of *MaPIP1-7*, *MaPIP2-6*, and *MaPIP2-10* [64]. Li et al. isolated three cDNAs encoding PIPs from a cotton root cDNA library, named *GhPIP2;2*, *GhPIP2;1*, and *GhPIP1;1*, while Northern blot analysis showed that these three genes were preferentially expressed in young roots. Young dividing or elongating root cells in early root development were the main ones in which *GhPIP1;1*, *GhPIP2;1*, and *GhPIP2;2* were expressed. Real-time fluorescent quantitative RT-PCR further showed that a high abundance of transcripts for these three genes accumulated at high levels in 3-day-old roots, but sharply decreased in roots aged 6-14 days during seedling development, indicating the developmental regulation of the isolated *GhPIP* genes in roots [60]. The results of real-time fluorescent quantitative PCR indicated that the expression of two *CsPIPs* genes exhibited certain tissue specificity, with *CsPIP2;7* having the highest expression level in leaves, and *CsPIP2;8* being expressed in flowers and leaves [117]. Wang Fang first identified the immunohistochemical tissue localization of the glycyrrhizin membrane water channel protein *GuPIP1* in licorice, showing strong expression in the epidermal cells of the root tip and root cap cells [118]. PIP family members are abundant, with varied expression in different tissues within plants, indicating the important roles of PIP proteins at different stages and different parts of plant growth and development.

### 4.2. PIP Regulatory Mechanism

The post-translational modification of proteins is crucial for regulating protein activity, subcellular localization, protein stability, protein–protein interactions, and three-dimensional structure, thereby enabling them to function properly. The activity of plant PIP proteins is regulated by various mechanisms, such as phosphorylation, acetylation, methylation, glycosylation modifications, pH protonation, and divalent ions.

#### 4.2.1. Phosphorylation Modification

Protein phosphorylation is one of the most important post-translational modification methods of proteins, and can regulate the function of proteins by activating or inhibiting their activities in cell signaling. Studies have shown that the Ser residues of many AQPs in plants are phosphorylated. Through protein phosphorylation proteomics analysis, the accumulation of multiple OsAQPs proteins was detected on the plasma membrane and vacuolar membrane of rice roots and shoots, with phosphorylation sites identified on *OsPIP2;1*, *OsPIP2;6*, and *OsPIP2;7*; similarly, multiple phosphorylation sites were identified on several PIPs of *Arabidopsis* and barley [119,120,121,122]. In *Arabidopsis*, 14-3-3 proteins can interact with the AtPIP2;1 protein and phosphorylate its Ser^280^ and Ser^283^ at the C-terminus, activating the transport activity of *AtPIP2;1*, which can regulate leaf water permeability under dark conditions and participate in the regulation of photosynthesis [123]; the cytoplasmic receptor kinase LP2 can interact with *OsPIP1;1*, *OsPIP1;2*, and *OsPIP1;3*, possibly regulating rice’s response to drought stress through phosphorylation mechanisms [124]. In vivo and in vitro experiments confirmed that the Ser residues of ZmPIPs proteins are phosphorylated by calcium-dependent protein kinases, with the phosphorylation sites of ZmPIP2s and ZmPIP1s located at the C-terminus and N-terminus, respectively; phosphorylation at the Ser^126^ or Ser^203^ sites of ZmPIP2;1 enhances its permeability to water [125]. The phosphorylation level of plant AQPs is also regulated by various factors. The phosphorylation level of AtPIPs proteins in *Arabidopsis* roots is regulated by NaCl and H_2_O_2_: the phosphorylation level of Ser^283^ of AtPIP2;1 decreases by 30% and affects its subcellular localization pattern during NaCl treatment, while its dephosphorylation level increases by 20% during H_2_O_2_ treatment; ABA treatment leads to a reduction in the phosphorylation level of some AtPIPs [121,126], and ethylene and the ethylene response factor EIL1/EIN3 participate in the phosphorylation of Ser^280^ and Ser^283^ at the C-terminus of AtPIP2;1, regulating water channel activity [127].

#### 4.2.2. Other Proteins Translated Post Modification

Researchers analyzing the stability of cauliflower vacuoles found that multiple BoPIPs undergo protein acetylation under high salt conditions, which may involve the permeability of vacuoles to water and the stability of membrane proteins [128]. Santoni et al. analyzed the co-translational and post-translational modifications of AtPIPs proteins in Arabidopsis roots using high-resolution mass spectrometry and found that the translation initiation amino acid Met of AtPIP2s can be acetylated or cleaved, AtPIP1s can be N-acetylated; in addition, multiple amino acid residue sites of AtPIP2s can be methylated [129]. The N-terminal Glu residue of AtPIP2;2 is a methylation site [130].

#### 4.2.3. Other Regulatory Mechanisms

Many AQP transport activities are inhibited by HgCl_2_, such as the inhibition of water permeability by HgCl_2_ on OsPIP1;1 [131]. The divalent cation Hg^2+^ can induce the pancreatic enzyme to hydrolyze the 33 kDa sugar beet BvPIP protein into 22 kDa [132]. Crystal structures show that Hg^2+^ ions can bind to the Cys^91^, Cys^127^, and Cys^132^ sites of SoPIP2;1; and the conserved Cys residues in the LA loop of PIP1s are the sensitivity sites of Hg^2+^ [133,134]. Divalent cations Ca^2+^ and Cd^2+^ inhibit the ion permeability of AtPIP2;1, with Asp^28^ and Glu^31^ of SoPIP2;1 being Cd^2+^ binding sites; in yeast, the transport of H_2_O_2_ by AtTIP1;1 is inhibited by Ag^2+^ [135,136]. Decreasing the cytoplasmic pH will reduce the water permeability of strawberry FaPIP2;1; the phosphorylation status of Ser^121^ and Ser^131^ on the LB loop of FaPIP2;1 influences its pH and regulates its sensitivity to the cytoplasmic pH environment [137]; the His^196^ site of NtPIP2;1 is a key site for the pH-dependent regulation of its transport activity [138,139].

## 5. Outlook

The AQP protein family is crucial for the transport of water and solutes under various biological stresses such as cold, salt, heavy metals, and drought. The physiological function of PIPs requires multiple mechanisms to work together. PIPs have been identified and named in various plants such as maize, rice and Arabidopsis. However, many questions regarding PIP genes remain to be further investigated. For example, (1) researchers have conducted gene family analysis of PIPs in many crops, but the functions of individual PIP genes are yet to be fully understood. Additional research is required to comprehensively explain the potential mechanisms of PIPs in plant tolerance to abiotic stresses. In-depth studies are needed to explore crosstalk regulation among different PIP and other MIP members, signaling molecules, and plant growth regulators, and their roles in abiotic stress. Using omics approaches, such as metabolomics, proteomics, and transcriptomics, in non-biological stress-tolerant plants can reveal the mechanisms of PIPs and their link to tolerance to environmental stresses. Therefore, PIP genes hold great promise as candidate genes in plant–pathogen interactions, and deciphering their mechanisms of action is crucial. (2) PIPs can interact with NIPs, members of the AQP family, to regulate water diffusion cooperatively [140]. It is worth further investigating whether PIPs interact with other members of the AQP family, such as SIPs and TIPs. (3) The expression, abundance, and transport activity of PIPs are tightly regulated at multiple levels, but further research is needed to decipher the molecular mechanisms involved in transcriptional, post-transcriptional regulation, and molecular interactions. Understanding how PIPs participate in endocytosis and how effector molecules pass through membrane transport could have significant implications. With the booming development of bioinformatics, genomics, proteomics, molecular biology, and other interdisciplinary fields, a more systematic analysis of the transcriptional regulatory network and protein modification mechanisms mediated by PIP genes in plant growth, development, and stress response is expected to provide valuable gene resources and scientific reference for the genetic improvement of crops.

## Figures and Tables

**Table 1:** The key differences between the PIP1 and PIP2 subgroups.

		PIP1	PIP2	Reference
Structure	C-terminal extension	Shorter	Longer	[17]
	N-terminal extension	Longer	Shorter	[17]
Function	Water channel activity	Lower	Higher	[15]
Distribution and gene numbers of different species	*A. thaliana*	5	8	[19]
	*O. sativa*	3	8	[20]
	*C. arietinum*	4	5	[21]
	*C. sinensis*	4	7	[22]
	*Phaseolus vulgaris*	5	7	[23]
	*Coffea canephora*	3	4	[24]
	*Medicago sativa*	5	5	[25]
	*Linum usitatissimum*	5	11	[26]
	*Zea mays*	6	7	[27]

**Table 2:** List of PIP genes involved in abiotic and biotic stresses.

Gene	Species	Research Methods	Stress Condition	Specific Functions	References
*AtPIP1;2*	*A. thaliana*	Transcription level	Drought	Downregulated in the roots	[63]
			Salt ion	Upregulation in the roots	[63]
*MaPIP2-3* *MaPIP2-7*	*Musa paradisiaca*	Transcription level	Drought	Upregulation in the Baxijiao and Fenjiao	[64]
*MaPIP2-6*	*Musa paradisiaca*	Transcription level	Drought	Reduced the drought resistance of Baxijiao and improved the drought resistance of Fenjiao	[64]
		Overexpression in transgenic banana	Salt ion	Increased resistance to salt stress	[82]
*MaPIP1;1*	*Musa paradisiaca*	Overexpression in Arabidopsis	Drought	Improved ion distribution, reduced membrane damage, and increased Arabidopsis resistance to drought stress	[68]
*MusaPIP1;2*	*Musa paradisiaca*	Overexpression in transgenic banana	Drought	Improved cell water levels and improved drought resistance of transgenic banana plants	[67]
			Low temperature	Positively influence tolerance to cold stress	[67]
*TaAQP8*	*Triticum aestivum*	Overexpression in tobacco	Drought	Increased tobacco resistance to drought stress	[69]
*OsPIP2;2*	*O. sativa*	Overexpression in rice protoplasts	Drought	Enhanced H_2_O transport and drought responses	[70]
*SlPIP2;1, SlPIP2;7, SlPIP2;5*	*Solanum* *lycopersicum*	Transcription level	Drought	Improved water uptake by maintaining osmotic balance and improving water content	[71]
*AtPIP2;5*	*A. thaliana*	Transcription level	Low temperature	Upregulated	[63]
*OsPIP1;1 OsPIP1;2*	*O. sativa*	Transcription level	Low temperature t	Increased resistance to cold tolerance	[72]
*MdPIP2;5a MdPIP2;5b*	*Malus domestica*	Overexpression in Arabidopsis	Low temperature	Increased the tolerance of transgenic Arabidopsis to cold stress	[73]
			Salt ion	Increased the tolerance of transgenic Arabidopsis to salt ion stress	[73]
*PaPIP1-2*	*Prunus armeniaca*	Overexpression in Arabidopsis	Low temperature	Enhanced the growth of transgenic plants under cold stress by lowering the level of MDA, increasing Pro accumulation, and increasing SOD activity	[74]
*RcPIP2;1 RcPIP2;2*	*Rhododendron* *catawbiense*	Overexpression in Arabidopsis	Low temperature	Lower ability to resist freeze drying	[75]
*TaAQP7*	*T. aestivum*	Overexpression in tobacco	Low temperature	Stronger cold tolerance for transgenic tobacco compared to non-transgenic tobacco	[77,78]
*OsPIP1;1*	*O. sativa*	Overexpression in transgenic rice	Salt ion	Enhanced salt tolerance	[81]
*TaPIP1;5*	*T. aestivum*	Transcription level	Salt ion	*MiR1118* primarily regulates membrane damage, ion homeostasis, and wh eat water status through *TaPIP1;5*	[83]
*CsPIP1;1*	*C. sinensis*	mRNA expression analysis	Salt ion	Significant upregulation in roots	[86]
*CmoPIP1-4*	*Cucurbita moschata*	Overexpression in yeast	Salt ion	Conferred salt tolerance to yeast	[84]
*ZmPIP1;1*	*Z. mays*	Overexpression in Arabidopsis	Salt ion	NaCl treatment induced *ZmPIP1;1* expression in roots and leaves, transgenic Arabidopsis plants also exhibited enhanced tolerance to salt stress.	[85]
*VvPIP2;1*	*Vitis vinifera*	Transcription level	Salt ion	Increased the transcription of *VvPIP2;1*	[87]
*EsPIP2;1*	*Eutrema salsugineum*	Overexpression in Arabidopsis	Salt ion	Enhanced the salt stress tolerance of Arabidopsis	[89]
*CfPIP2;1*	*Cucurbita ficifolia*	Overexpression in Arabidopsis	Salt ion	Increased the germination rate of seeds subjected to high salt stress	[90]
*HvPIP2;1*	*Hordeum vulgare*	Overexpression In transgenic rice	Salt ion	Decreased salt tolerance	[91]
*HvPIP1;3* *HvPIP1;4*	*H. vulgare*	Overexpression in yeast	Pseudo metal ion (B)	Conferred boron transport function	[92]
*AtPIP1;2* *AtPIP2;1 AtPIP2;2*	*A. thaliana*	Transcription level	Pseudo metal ion (B)	Reduced water flow to the shoots, prevented excess boron accumulation in plant tissues	[93]
*OsPIP2;6*	*O. sativa*	Overexpression in Arabidopsis and RNAi-mediated approach	Pseudo metal ion (B and As)	Yielded high boron tolerance in transgenic Arabidopsis and transported of arsenic from roots to stems, reduced arsenic accumulation in rice	[95]
*OsPIP1;3* *OsPIP2;4 OsPIP2;7*	*O. sativa*	Transcription level	Pseudo metal ion (B and As)	Involved in the transport of boron arsenate (As III) and provided tolerance to As(III) and boron toxicity	[96,97]
*AtPIP2;2*	*A.thaliana*	Overexpression in Arabidopsis and yeast	Pseudo metal ion (As)	Increased the tolerance of yeast and Arabidopsis overexpressing As(III), reduced As(II) levels in yeast	[98]
*AtPIP1;4 AtPIP2;4*	*A. thaliana*	Overexpression in Arabidopsis	Plant diseases	Enhanced resistance to *Pseudomonas syringae*	[105,106]
*OsPIP2;2*	*O. sativa*	Protein interaction	Plant diseases	By regulating *OsmaMYB* nuclear translocation to inhance the resistance to bacterial blight, sheath blight, and blast disease.	[110]
*OsPIP1;2*	*O. sativa*	Protein interaction	Plant diseases	Suggested its involvement in rice’s immune response to *Xanthomonas oryzae* pv. *oryzae*	[111]

## Data Availability

The materials generated in this study are available from the corresponding author upon request.
